# Targeting cyclic nucleotides in CNS drug discovery: a translational approach into cognition enhancement

**DOI:** 10.1186/2050-6511-16-S1-A18

**Published:** 2015-09-02

**Authors:** Jos Prickaerts

**Affiliations:** 1 Department of Psychiatry and Neuropsychology, School for Mental Health and Neuroscience, Maastricht University, Maastricht, The Netherlands

## 

Cyclic adenosine monophosphate (cAMP) and/or cyclic guanosine monophosphate (cGMP) have been suggested to play specific roles in processes of memory. These cyclic nucleotides are hydrolyzed by specific enzymes, i.e. phosphodiesterases (PDEs). Thus, selective PDE inhibitors preventing the breakdown of cAMP and/or cGMP could improve memory. Studies with different timing of treatment with specific PDE inhibitors indicated that distinct underlying signaling pathways for early and late consolidation processes exist corresponding to specific time-windows for memory consolidation into long-term memory. There is evidence that the underlying mechanisms of PDE inhibition on the observed behavioral effects are independent of possible cerebrovascular effects. Most likely the underlying mechanisms are a cGMP/PKG pathway for early consolidation processes and a cAMP/PKA pathway for late consolidation processes. In addition, the early-phase cGMP/PKG signaling actually requires late-phase cAMP/PKA-signaling in long-term memory formation. Recently, the effects of specific PDE inhibitors are explored on other cognitive domains including acquisition processes/short-term memory and information processing. It will be shown that elevation of central cGMP levels or cAMP levels after treatment with a specific PDE inhibitor both improve acquisition processes/short-term memory. The effects of specific PDE inhibitors on information processing by using a sensory gating paradigm indicate that elevation of cGMP together with cAMP using a specific PDE inhibitor improves basic information processing, whereas elevation of cGMP alone has no effect. In a translational approach we also investigated the effect of PDE5 inhibition on cognition in humans. However, in contrast to studies including rodents and monkeys, PDE5 inhibition had no effect on cognition including memory processes in humans. Yet as sensory gating was also not affected this could underlie the translational value of this measure. It is clear that the transition of a drug from preclinical to clinical creates translational hurdles. Within the context as described above, the latest results of specific PDE inhibitors on cognitive processes will be presented and its implications will be discusses for finding and testing new cognition enhancers.

